# Prevalence, risk factors and perceptions of caregivers on burns among children under 5 years in Kisenyi slum, Kampala, Uganda

**DOI:** 10.1186/s40621-022-00382-w

**Published:** 2022-06-10

**Authors:** Marcia Tusiime, David Musoke, Fiston Muneza, Milton Mutto, Olive Kobusingye

**Affiliations:** 1 Department of Disease Control and Environmental Health, School of Public Health, Makerere University College of Health Sciences, Kampala, Uganda; 2 Chronic Trauma, Injury and Disability Program (TRIAD), School of Public Health, Makerere University College of Health Sciences, Kampala, Uganda; 3 Pincer Training and Research Institute, Kampala, Uganda

**Keywords:** Burns, Prevalence, Factors associated, Risk perception, Informal settlement, Uganda

## Abstract

**Background:**

Globally, burn related deaths are disproportionately higher among children below 5 years of age compared to other age groups. Although rarely fatal, most burns in this group occur within homes specifically in kitchens. This study assessed the prevalence, risk factors and perceptions of caregivers regarding burns among children under 5 years in an urban slum in Kampala, Uganda.

**Methods:**

The study used an analytic cross-sectional design with quantitative and qualitative techniques. Quantitative data were collected using a structured questionnaire and observational checklist, while qualitative data involved use of a key informant interview guide. A total of 426 children were involved in the study, while 6 key informants namely an adult mother, teenage mother, community health worker, health practitioner, father and local leader were interviewed. A modified Poisson regression model was used to determine the correlates of burn injuries, prevalence rate ratios and 95% confidence intervals, while thematic analysis was used for qualitative data.

**Results:**

The prevalence of burns among under-fives was 32%, highest among those aged 24 to 35 months (39%), and least in those below 12 months (10%). Children with single parents (adj PR = 1.56 95% CI 1.07–2.29) and those from households in the middle and least poor wealth quintile (adj.PR = 1.72; 95% CI 1.02–2.89 and adj.PR = 1.77; 95% CI 1.02–3.05, respectively) were more likely to get burns compared to their counterparts in other quintiles. In households where flammables were safely stored, children were less likely to suffer from burn injuries (adj.PR = 0.61; 95% CI 0.44–0.83). Congestion, negligence of caregivers, and use of charcoal stoves/open cooking were the commonest determinants of burns. Although many caregivers offered first aid to burn patients, inadequate knowledge of proper care was noted. Crawling children were perceived as being at highest risk of burns.

**Conclusion:**

The prevalence of burns among children under 5 years was high, with several household hazards identified. Health education, household modification and applicable public health law enforcement are recommended to reduce hazards and minimise burn risks among children.

## Background

Burns are a global public health problem, accounting for an estimated 180 000 deaths annually. The majority of these occur in low- and middle-income countries and almost two thirds occur in the WHO African and South-East Asia regions (WHO 2018). Over 1 million burns occur in the African Region (SSA) each year leading to significant morbidity and mortality (Collier et al. 2021). Children who are below 4 years have disproportionately higher mortality compared to the other age groups (Forjuoh 2006; Peck et al. 2008). Statistics on burns among under-fives in slums remain scanty, undocumented or unpublished. Without precise data on prevalence and associated factors, the design of home-based burn prevention interventions is greatly hampered. Although rarely fatal, 85% of burn injuries are due to scalds (Fleisher and Ludwig 2010; Lee 2011), and 95% of them occur within the home environment (Lee 2011) specifically the kitchen (Mashreky et al. 2010; Kumar et al. 2000). These burns leave long term consequences such as pain, disfigurement, emotional trauma, disability and in extreme cases death. In African region, burns account for a high proportion of injury deaths although its magnitude is hardly documented (Forjuoh 2006).

Homes are among the leading injury locations, with children living in informal settlements being at higher risk of burns. Infants are exposed to hazards which are often difficult to eliminate especially in informal settlements and are characterised by congestion and overcrowding (Outwater et al. 2013; Mashreky et al. 2010). Given the adventurous and inquisitive nature of children, the lack of designated safe play spaces further pre-disposes them to burn injuries. With most burn studies focusing on all age groups and relying on hospital data from across the globe (Peden 2008; Vendrusculo et al. 2010; Ndiritu et al. 2006; Mzezewa et al. 2000; Butchart et al. 2000), there is insufficient evidence to attribute specific factors to increased risk of burns among under-fives at household level in Uganda.

Injury studies in Uganda are generally biased towards a specific study population or geographic scope, while most published literature is on road traffic injuries (RTIs) among persons of older age (Kiguli et al. 2005). In addition, emphasis has mainly been put on RTIs reported at the emergency departments of hospitals hence underrepresenting injuries occurring at household level especially among children under 5 years of age. Based on the iceberg principle, only severely injured children report to the hospital which implies that many injuries are treated at home hence the need for a community survey. The study therefore sought to establish the prevalence of burns among children under 5 years at household level in Kisenyi slum-in Uganda together with the associated risk factors. In addition, the perceptions of caregivers on childhood burns were investigated as they play key role in burn prevention and treatment within homes.

## Methods

### Study design and setting

A cross-sectional study was conducted in Kisenyi II slum in the Central Division of Kampala City, Uganda using quantitative and qualitative techniques. Kisenyi II is a highly populated residential area with a population of nearly 9000 inhabitants with primarily many small-scale businesses as the main income source for residents. At the time of the study, Kisenyi was the most populated zone in Kampala’s Central Division with nearly 8,200 residents. The area has many maize mills and the residents also rear goats and sell fresh fruits and vegetables. Most dwellers subsist by vending food, meat, general merchandise and recycling. Kisenyi mainly has permanent houses although a few are mud and wattle, or wood and plastic.

### Sample size and sampling procedure

A sample of size of 426, was determined using Daniel (1999) formula design effect of 1.2 to compensate for inter-strata difference. A stratified random process was used to select participating households from all 10 zones in Kisenyi II parish. The sampling frame was drawn by Community Health Workers. The principal investigator used the table of random numbers to generate the sample. The consenting adult found at home at the interview was included in the study. Where there was more than one eligible child below 5 years in the household, the one with the most recent injury was considered. If the selected child had suffered multiple injuries in the past, the most recent burn injury was considered for the study. Time frame was a burn injury that occurred within the last 1 year (recall period based on previous related studies). Multiple burn injury data were excluded from analysis. Children with no burns were included in determining prevalence as they constituted the denominator.

### Study participants and data collection

The target population was children aged 0–5 years in Ugandan slums and the accessible population was those Kisenyi. The caregivers were used as proxies. Households that did not respond or those where caregivers were inaccessible were excluded after three attempts. The main outcome was a burn injury in the last 1 year. Independent variables included sex, age, previous burn, presence of burn scar, pre-existing impairment, burn outcome; care giver occupation, age, educational level, marital status and alcohol/tobacco abuse, storage of 3flammable substances, use of electric appliances, source of lighting, source of cooking energy, presence of loose clothing, reliability of water supply, asset ownership, type of housing structure, separation of cooking area, presence of playing area, crowding, accommodation type, and house occupancy. Six key informant interviews were conducted to explore caregivers’ perceptions of risk and of causes and management of burns. These included adult mother, teenage mother, community health worker, health practitioner, father and local leader. They were selected purposively based on previous injury studies. During that during data collection, now newer information was obtained hence reaching saturation after 6 interviews. Triggering questions included: perceived risk of burn injury in regard to magnitude of burns in study community, causes of childhood burns, reasons for common child burn injury, cultural beliefs about burn treatment and care, burn prevention and safety measures in place, and proposed design measures to increase childhood safety from burns within the community. The key informants were interviewed by trained research assistants who were recruited based on prior research experience in similar settings (slum areas). These Research Assistants, who all had undergraduate degrees, were trained by the research team prior to the study so as to understand the study objectives, study tool pre-test and data collection methods. The data collection tools were pretested in a different location within Kampala. The quantitative questionnaire was translated into *Luganda*, the main local language used in the area. An observation checklist was also used to assess the physical environment within each household. Qualitative data were recorded using a handheld voice recorder, transcribed, translated from local language to English and analysed manually to develop codes and themes.

### Data analysis

Quantitative data were analysed using Stata Version 13. Categorical variables were summarized as proportions while continuous variables as means. The relationship between independent variables and burn injury was assessed using prevalence rate ratios (PRR) based on modified Poisson regression modelling (Barros and Hirakata 2003). Prevalence Ratios (PR) were preferred to Odds Ratios (OR) because of the high prevalence of the outcome (> 10%) in the area since it provides a better risk estimate than the OR (Thompson et al. 1998; Barros and Hirakata 2003). Congestion was deemed to occur where more than 4 adults live in a single roomed house. Principal component analysis (PCA), a multivariable reduction method, was used to create a wealth quintile to measure socio-economic status of the household. Assets included in the PCA were: electricity, radio, television, computer, fridge, watch, mobile phone, bicycle, motorcycle, pushcart, car, boat and bank account ownership. Thematic analysis was used manually for the qualitative data which involved generation of codes, and later themes. Due to the relatively small number of interviews conducted hence Nvivo was not utilised.

### Ethical considerations

The study was approved by the Makerere University School of Public Health Higher Degrees, Research and Ethics Committee. Permission was also granted by the Director of Public Health at Kampala Capital City Authority, and Local Council 1 chairpersons of the zones in Kisenyi parish involved in the study. Written informed consent was obtained from the caregivers on behalf of the children before they participated in the study.

## Results

### Demographic characteristics

Of the 426 children recruited into the study, 223 (52.4%) were boys, and the mean age was 2.94 years (SD = 1.29). Most (70.2%) injured children were between 2 and 4 years old. The majority (91.8%) of caregivers who answered on behalf of the children were women, while 62.0% of them had a median age of 29.5 months. The majority (89.7%) of respondents were biological mothers to the children, while 23% had no formal education. Most (91.3%) of the household heads were self-employed, while many of the children (67.4%) lived with married parents (Table 1).

**Table 1:** Background characteristics of respondents

Variable	N = 426 (%)
Sex	
Male	223 (52.4)
Female	203 (47.7)
Age of child (months)	
0–11	74 (17.4)
12–23	92 (21.6)
24–35	98 (23.0)
36–47	109 (25.6)
48–60	53 (12.4)
Zone of residence	
Church area	72 (16.9)
Kakajjo	173 (40.6)
Kasaato	62 (14.6)
Kiganda	119 (27.9)
Caregiver's sex	
Male	35 (8.2)
Female	391 (91.8)
Caregivers’ age (years)	
< 29.5	264 (61.9)
≥ 29.5	162 (38.03)
Caregivers’ relationship to child	
Mother	382 (89.7)
Other relative	44 (10.3)
Caregivers’ education status	
None	100 (23.5)
Primary	157 (36.9)
Secondary and above	169 (39.7)
Smoking history of caregiver	
Never	43 (10.1)
Weekly	42 (9.9)
Daily	341 (80.1)
Alcohol use by caregiver	
Never	302 (70.9)
Weekly	63 (14.8)
Daily	61 (14.3)
Occupation of household head	
Self employed	391 (91.8)
Unemployed	35 (8.2)
Marital status of household head	
Married	287 (67.4)
Widowed	89 (20.9)
Single	50 (11.7)
Religion of household head	
Anglican	54 (12.7)
Pentecostal	42 (9.9)
Catholic	176 (41.3)
Muslim	154 (36.2)
Ethnicity	
Central	164 (38.5)
Eastern	58 (13.6)
Migrant	59 (13.9)
Northern	59 (13.9)
Western	86 (20.2)

### Revalence of burns among children under 5 years and associated factors

The prevalence of burns in the study was 32%. The commonest (42.2%) cause of burns was hot liquids (scalds), while hot solids and fires caused 41% and 17%, respectively. Forty seven percent of burns occurred outdoors, while nearly half of them (44%) happened during the afternoon playtime. Children between 2 and 5 years were three times more likely to suffer burns (adj.PR = 2.9, 95% CI 1.6–5.5 and adj.PR = 3.8, 95% CI 2.04–7.1, respectively) in comparison with those below 1 year. Those under the care of single parents were 1.56 times more likely to suffer a burn injury (adj.PR = 1.56, 95% CI 1.07–2.29) while those born to migrants (adj.PR = 0.5, 95% CI 0.28–0.91) and those from less poor wealth quintile (adj.PR = 0.88, 95% CI 0.51–1.51) had lower risk of experiencing burn injuries. In households where flammables were safely stored, the likelihood of under-five burns was lower by 39% (adj.PR = 0.61, 95% CI 0.44–0.83) (Table 2).

**Table 2:** Factors associated with burns among under-fives in Kisenyi, Kampala

Variable	Burn history				Multivariable analysis	
	Yes		No		PR (95%CI)	*p* value
	n	%	n	%		
*Individual factors*						
Age of child (in months)						
0–11	10	7.41	64	21.99	1	
12–23	24	17.78	68	23.37	2.1 (1.1–4.02)	0.024**
24–35	39	28.89	59	2..27	3.4 (1.92–6.28)	< 0.001**
36–47	36	26.67	73	25.09	2.6 (1.45–4.94)	0.002**
48–60	26	19.26	27	9.28	3.8 (2.04–7.07)	< 0.001**
Zone						
Church area	18	13.33	54	18.56	1	
Kakajjo	71	52.59	102	35.05	1.7 (1.12–2.85)	0.014**
Kasaato	17	12.59	45	15.46	1.2 (0.73–2.22)	0.379
Kiganda	29	21.48	90	30.93	1.01 (0.6–1.71)	0.957
Caregivers’ sex						
Male	5	3.7	30	10.31	1	
Female	130	96.3	261	89.69	2.4 (1.08–5.38)	0.031**
Caregivers’ education status						
None	32	23.7	68	23.37	1	
Primary	58	42.96	99	34.02	0.98 (0.65–1.48)	0.956
Secondary and above	45	33.33	124	42.61	0.77 (0.51–1.16)	0.216
Alcohol use by caregiver						
Never	90	66.67	212	72.85	1	
Weekly	18	13.33	45	15.46	0.91 (0.59–1.39)	0.674
Daily	27	20	34	11.68	1.28 (0.92–1.78)	0.136
Occupation						
Employed	121	89.63	270	92.78	1	
Unemployed	14	8.22	21	7.22	1.20 (0.76–1.87)	0.399
Marital status						
Married	82	60.74	205	70.45	1	
Widowed	30	22.22	59	20.27	1.25 (0.88–1.78)	0.207
Single	23	17.04	27	9.28	1.56 (1.07–2.29)	0.020**
Religion						
Anglican	12	8.89	42	14.43	1	
Pentecostal	16	11.85	26	8.93	1.25 (0.66–2.35)	0.479
Catholic	62	45.93	114	39.18	1.55 (0.88–2.7)	0.123
Muslim	45	33.33	109	37.46	1.38 (0.76–2.52)	1.282
Ethnicity						
Central	60	44.44	14	35.74	1	
Eastern	17	12.59	41	14.09	0.88 (0.55–1.4)	0.611
Migrant	10	7.41	49	16.84	0.5 (0.28–0.91)	0.025**
Northern	18	13.33	41	14.09	0.81 (0.52–1.27)	0.370
Western	30	22.22	56	19.24	0.87 (0.62–1.22)	0.443
Wealth quintile						
Poorest	25	18.52	64	21.99	1	
Poor	35	25.93	76	26.12	1.10 (0.68–1.91)	0.616
Middle	25	18.52	33	11.34	1.72 (1.02–2.89)	0.040**
Less Poor	25	18.52	82	28.18	0.88 (0.51–1.51)	0.657
Least Poor	25	18.52	36	12.37	1.77 (1.02–3.05)	0.040**
*Environmental factors*						
Location of cooking area						
In house	44	32.59	130	44.67	1	
In separate building	6	4.44	12	4.12	1.50 (0.76–3.11)	0.223
Outdoors	85	62.96	149	51.2	1.36 (0.98–1.88)	0.065
Overcrowding						
No	34	25.19	93	31.96	1	
Yes	101	74.81	198	68.04	1.18 (0.86–1.62)	0.291
Cooking area accessible to child						
No	8	5.93	30	1.31	1	
Yes	127	94.07	261	89.69	1.67 (0.98–2.83)	0.058
Open fire used for cooking						
No	70	51.85	134	46.05	1	
Yes	665	48.15	157	53.95	0.76 (0.57–1.01)	0.062
Kitchen separate from other room						
No	111	82.22	217	74.57	1	
Yes	24	17.78	74	25.43	0.75 (0.49–1.15)	0.197
Flammables safely stored						
Yes	52	38.52	83	61.48	1	
No	93	31.96	198	68.04	0.61 (0.44–0.83)	0.002**

**Statistically significant at p < 0.05; adj.PR = adjusted prevalence ratio

From the qualitative data, four major themes were identified concerning risk perceptions among caregivers: commonest cause of burns, most affected age group, burn treatment methods, and proposed control measures. From the interviews, the main causes of burns were overcrowding, use of open cooking stoves without demarcated cooking areas, caregiver negligence, and limited space for multiple purposes.. Most caregivers offered home remedies as first aid to children with burns which varied from aloe vera plant sap, flour, urine, sugar, burnt sack, and toothpaste. Treatment at health facilities was considered last resort when the local remedies fail. The key informants proposed measures to reduce burn related childhood risks including enforcement of bye-laws on safe and cleaner lighting and cooking options such as solar, having raised cooking places, having gazetted cooking places, discouraging candle use, and periodic community health education. One teenage mother said:

“Even at home children get these burns from the charcoal stove, because as I said that we live in a congested environment, our houses are crowded, you cook in a place where these children play from, so they end up falling in the fire.” Teenage mother, Kakajjo zone

The caregivers largely depended on home remedies to administer first aid to burn injuries, with medical care sought as a last resort. One respondent said:

“When a child gets burns, what we first do, and what we grew up seeing, is to first smear this child with cooking oil, then sugar. If we see that the condition is serious and it needs a health facility, we take the child to the Kampala Capital City Authority health facility for further management.” Local leader, Kakajjo zone

Among the proposed burn prevention measures were use of safe energy sources just as one mother put it:

“If possible, I would encourage people to use solar energy because this reduces the risk of burns among children compared to the candles in our homes”. Adult mother, Church zone

## Discussion

Caregivers perceived congestion, negligence of caregivers, and use of charcoal stoves/open cooking places to be among the commonest causes of burns. Low knowledge on first aid and proper burn treatment and care among caregivers was also noted, while children of crawling age were perceived as being at highest risk of burns. The findings of this study highlight the public health risks associated with childhood injuries that emerge from living in slum settings. The recommendations from the study can inform the design of future public health education and physical urban planning programmes to ensure the health and safety of children at households.

The prevalence of childhood burns in this study was 32% which was inconsistent with earlier findings which documented much lower prevalence of burns. Indeed, the prevalence of childhood burns in the other studies ranged from 5 to 11% in rural Ethiopia (Forjuoh et al. 1995a), and 6% amongst Ghanaian children (Courtright et al. 1993). Given these studies were conducted more than a decade ago, and the rampant rural–urban migration could possibly explain why the prevalence of burns in our study could have more than doubled among under-fives (Forjuoh et al, 1995a, 1995b). It is also worth noting that most other community studies document incidence of burns or fire specifically with study areas varying from home to health facilities (Kobusingye et al. 2001; Chen et al. 2007; Mutto et al. 2011; Wong et al. 2014) which is different from our study. Given that our study was conducted in a slum, the set up in such an environment could also proliferate occurrence of accidents including burns hence the high prevalence observed. Therefore, other studies to further explore the high prevalence of burns in slums in comparison with other settings are needed.

2Our study found the child’s age was significantly associated with burns which was similar to results from other studies which mentioned that the likelihood of burns increased with age, and that children aged 2–4 years had the highest risk (Peck et al. 2008; Flavin et al. 2006; Shah et al. 2013; Barnes and Moiloa 2004; Gali et al. 2004; Ndiritu et al. 2006).ch. Child health programmes could target use of demarcated play spaces for children living in slums, coupled with continued sensitisation of child caregivers on injury hazards. These measures can be done through existing health, nutrition and sanitation messages given at health care facilities, or by health partners working within slum communities.

Children whose caregivers were single were two times more likely to have burns as compared to those living with both parents. This finding is similar to other studies that mention that households with couples offer more protection to children as it is linked with better supervisory practices (O'Connor et al. 2000; Khandarmaa et al. 2012; Shah et al. 2013; Edelman 2007; Tiikkaja et al. 2009; Pearce et al. 2012; Howe et al. 2012). This is linked to the fact that caregivers are usually the only adult supervising many children within the day in addition to other household chores. This is not only exhausting but leaves little or no room for appropriate childcare supervision. This situation often results in children spending the day unsupervised by the adult hence their increased exposure to numerous injuries including burns. Therefore, children living with more adults in a given household are likely to be better supervised and protected from burns than those with a single person.

Regarding socioeconomic status, findings from our study showed that children from wealthier households were more likely to suffer burns as compared to those from poorest households which is contrary to many studies which found that children from informal settlements were at higher risk of injury (Butchart et al. 2000; Bartlett 2002). Unlike many studies conducted in African region which link ethnic minority groups and immigration status to increased risk of burns (Edelman 2007; Vendrusculo et al. 2010; Shah et al. 2013; Kamal 2013; Van Niekerk et al. 2006), in our study the risk of burns was significantly reduced for children of migrants. This finding can be possibly linked to the fact that migrant families tend to settle in large numbers within a given household which offers increased adult supervision for children under 5 years hence the reduced burn risk. This could further add to evidence that adult supervision plays a significant role in reducing exposure to burn injuries among under-fives. However, more studies are needed to further explore the reduced risk of burns among children of migrants.

In our study, there was reduced burn injury risk significantly among under-five children who resided in houses that were observed to have separate storage for flammables. Literature confirms the association between safe storage of flammables and burn injury likelihood (Forjuoh 2006; Mzezewa et al. 2000; Vilasco and Bondurand 1995) which is in line with our findings. This therefore implies that ease of access to flammable substances by children increases risk of burn injuries. However, it should be noted that due to the limited space in slums, areas dedicated solely for storage of flammables may be rare compared to other settings. Proper storage of flammable substances within households should therefore be pointed out during health education campaigns to caregivers and the general population to reduce the risk of child burns.

It is worth noting that there was no statistically significant difference in child burn injury risk among households which had a raised cooking place compared to those that lacked one. This finding was synonymous with a Ugandan study by Mutto et al. (2011) which also found that use of a raised cooking place was not associated with burn incidence. Therefore, even as other studies (Outwater et al. 2013; Barnes and Moiloa 2004) have found the use of a raised cooking place to reduce risk of burns in Kisenyi and other urban slum areas, this may not always be the case. It is logical that having a raised fireplace which limits access of children to cooking would reduce the risk of burns. However, the lack of statistical significance between a raised cooking place and burns in our study could be an indication that these incidents could be happening beyond the cooking process. For example, children could get burns from hot water left on the floor following cooking it. Therefore, it is important to look at other factors that could lead to burns among children beyond the cooking place.

Management of burns given the strong belief in the efficacy of traditional remedies among the key informants. Many acknowledged wide use of home remedies by slum dwellers with the belief that these can reduce burn severity, alleviate pain and prevent infection. Some studies have discussed the pros and cons of using traditional remedies in burn treatment but these belief still exist worldwide (Kopp et al. 2003; Karaoz 2020; Courtright et al., 1993; Ameh, 2002). These beliefs and myths may need to be demystified through community burn management education. Preventive measures specific to this context could include: community sensitization drives for burn injury prevention targeting single mothers, stay home mothers, house helpers and covering issues such as child supervision, safe storage practices for consumables, proper first aid and burn treatment, demystify myths and belief on burn management. These can be done by social workers and burn prevention professionals such as nurses with the aim of changing attitudes of people in regard to the importance of proper first aid and immediate medical attention to burn victims especially children. The enforcement of building regulations of the Public Health Act (PHA) stipulate that “*Every dwelling shall be provided with sufficient and suitable accommodation for cooking, storing food to the satisfaction of the local authority*” (PHA, part VI, article 64). These regulations should be enforced by health inspectors, city planners and the landlords within the study area, with hefty fines incurred by non-compliant households. Urban planners should be mobilized to create clear distinction between residential and commercial areas so as to reduce on potential burn injuries in slum areas. Demarcation and allocation of specific cooking and storage spaces for basic residential dwellings is being recommended to physical planners of urban authorities which can lower the number of burn injury cases in the community. Support uptake of clean energy solutions such as solar appliances for cooking and lighting, subsidized cooking stoves for households living in slum areas.

A limitation of this study is that self-reporting among caregivers could have resulted in recall bias. Nevertheless, this bias was minimised by use of the observational checklist to monitor some burn hazards within the home. Given that this was a cross-sectional study, other study designs may be used in future research to establish causality with numerous burn risk factors. The use of both quantitative and qualitative methods in our study offered increased scientific rigour hence a strength. In addition, that fact that this cross-sectional study was conducted at household level gives a more accurate estimate of burn injury prevalence compared to existing hospital-based burn injury studies which tend to under-estimate burn prevalence.

## Conclusion

This study revealed a high prevalence of burn injuries among under-fives in the urban slum setting. These injuries were significantly associated with: child factors such as age group, caregiver factors such as marital status, ethnicity, wealth quintile; and environmental factors such as storage method of flammables within the household. Interventions focusing on advocacy for appropriate modifications within the household such as use of solar power, enactment and enforcement of bye-laws on use of unsafe energy sources such as candles,, open fires for cooking, and development of health education messaging on education about prevention and pre-hospital care of home burn injuries targeting caregivers of under-fives is recommended.

## Data Availability

Data is available from the corresponding author on reasonable request.

## Abbreviations

OR: Odds ratios
PCA: Principal component analysis
PR: Prevalence ratios
PRR: Prevalence rate ratios
RTIs: Road traffic injuries

## References

[CR1] Ameh E. Burn statistics from Northern Nigeria. Paediatric Surgery Unit. 2002. Department of Paediatric Surgery, Ahmadu Bello University and Ahmadu Bello University Teaching Hospital.

[CR2] Barnes B, Moiloa K (2004). Domestic energy use, time activity patterns and risk of burns amongst children less than five years of age in rural South Africa: short research report. Afr Saf Promot.

[CR3] Barros AJ, Hirakata VN (2003). Alternatives for logistic regression in cross-sectional studies: an empirical comparison of models that directly estimate the prevalence ratio. BMC Med Res Methodol.

[CR4] Bartlett SN (2002). The problem of children's injuries in low-income countries: a review. Health Policy Plan.

[CR5] Butchart A, Kruger J, Lekoba R (2000). Perceptions of injury causes and solutions in a Johannesburg township: implications for prevention. Soc Sci Med.

[CR6] Chen G, Smith GA, Ranbom L, Sinclair SA, Xiang H (2007). Incidence and pattern of burn injuries among children with disabilities. J Trauma.

[CR7] Collier ZJ, Naidu P, Choi KJ, Pham CH, Potokar T, Gillenwater J. ., et al. 83 Burn Injuries in Sub-Saharan Africa: A Global Burden of Disease Study. Journal of Burn Care & Research 42. Supplement_1 (2021): S57-S58.

[CR8] Courtright P, Haile D, Kohls E (1993). The epidemiology of burns in rural Ethiopia. J Epidemiol Community Health.

[CR9] Edelman LS (2007). Social and economic factors associated with the risk of burn injury. Burns.

[CR10] Flavin MP, Dostaler SM, Simpson K, Brison RJ, Pickett W (2006). Stages of development and injury patterns in the early years: a population-based analysis. BMC Public Health.

[CR11] Forjuoh SN (2006). Burns in low- and middle-income countries: a review of available literature on descriptive epidemiology, risk factors, treatment, and prevention. Burns.

[CR12] Forjuoh SN, Keyl PM, Diener-West M, Smith GS, Guyer B (1995). Prevalence and age-specific incidence of burns in Ghanaian children. J Trop Pediatr.

[CR13] Forjuoh SN, Guyer B, Strobino DM, Keyl PM, Diener-West M, Smith GS (1995). Risk factors for childhood burns: a case-control study of Ghanaian children. J Epidemiol Community Health.

[CR14] Gali BM, Madziga AG, Naaya HU (2004). Epidemiology of childhood burns in Maiduguri north-eastern Nigeria. Niger J Med.

[CR15] Howe LD, Galobardes B, Matijasevich A, Gordon D, Johnston D, Onwujekwe O (2012). Measuring socio-economic position for epidemiological studies in low- and middle-income countries: a methods of measurement in epidemiology paper. Int J Epidemiol.

[CR16] Kamal NN (2013). Home unintentional non-fatal injury among children under 5 years of age in a rural area, El Minia Governorate. Egypt J Community Health.

[CR17] Karaoz B (2020). First-aid home treatment of burns among children and some implications at Milas. Turkey Journal of Emergency Nursing.

[CR18] Khandarmaa TO, Harun-Or-Rashid M, Sakamoto J (2012). Risk factors of burns among children in Mongolia. Burns.

[CR19] Kiguli S, Mutto M, Mukanga OD (2005). Unintentional home injuries among children under five years in a slum area of Kampala, Uganda: Prevalence and risk factors. East and Central African Journal of Surgery.

[CR20] Kobusingye O, Guwatudde D, Lett R (2001). Injury patterns in rural and urban Uganda. Inj Prev.

[CR21] Kopp J, Wang G, Horch R, Pallua N, Ge S (2003). Ancient Traditional Chinese Medicine In Burn Treatment: A historical review. Burns.

[CR22] Kumar P, Chirayil PT, Chittoria R (2000). Ten years epidemiological study of paediatric burns in Manipal. India Burns.

[CR23] Lee K, Marcdante KJ, Kliegman RM, Jenson HB, Behrman RE (2011). Burns. Nelson Essentials of Paediatrics.

[CR24] Mashreky SR, Rahman A, Khan TF, Svanström L, Rahman F (2010). Determinants of childhood burns in rural Bangladesh: A nested case-control study. Health Policy.

[CR25] Mutto M, Lawoko S, Nansamba C, Ovuga E, Svanstrom L (2011). Unintentional childhood injury patterns, odds, and outcomes in Kampala City: an analysis of surveillance data from the National Pediatric Emergency Unit. J Inj Violence Res.

[CR26] Mzezewa S, Jonsson K, Aberg M, Salemark L (2000). A prospective study of suicidal burns admitted to the Harare burns unit. Burns.

[CR27] Ndiritu S, Ngumi ZW, Nyaim O (2006). Burns: the epidemiological pattern, risk and safety awareness at Kenyatta National Hospital. Nairobi East Afr Med J.

[CR28] O’Connor TG, Davies L, Dunn J, Golding J (2000). Distribution of accidents, injuries, and illnesses by family type. ALSPAC Study Team. Avon Longitudinal Study of Pregnancy and Childhood. Pediatrics.

[CR29] Outwater AH, Ismail H, Mgalilwa L, Justin Temu M, Mbembati NA (2013). Burns in Tanzania: morbidity and mortality, causes and risk factors: a review. Int J Burns Trauma.

[CR30] Pearce A, Li L, Abbas J, Ferguson B, Graham H, Law C (2012). Does the home environment influence inequalities in unintentional injury in early childhood? Findings from the UK Millennium Cohort Study. J Epidemiol Community Health.

[CR31] Peck MD, Kruger GE, van der Merwe AE, Godakumbura W, Ahuja RB (2008). Burns and fires from non-electric domestic appliances in low and middle income countries Part I. The Scope of the Problem Burns.

[CR32] Peden M (2008). World report on child injury prevention appeals to "Keep Kids Safe". Inj Prev.

[CR33] Shah M, Orton E, Tata LJ, Gomes C, Kendrick D (2013). Risk factors for scald injury in children under 5 years of age: a case-control study using routinely collected data. Burns.

[CR34] Thompson ML, Myers JE, Kriebel D (1998). Prevalence odds ratio or prevalence ratio in the analysis of cross-sectional data: what is to be done?. Occup Environ Med.

[CR35] Tiikkaja S, Rahu K, Koupil I, Rahu M (2009). Maternal social characteristics and mortality from injuries among infants and toddlers in Estonia. J Epidemiol Community Health.

[CR36] Niekerk AV, Reimers A, Laflamme L (2006). Area characteristics and determinants of hospitalised childhood burn injury: a study in the city of Cape Town. Public Health.

[CR37] Vendrusculo TM, Balieiro CR, Echevarría-Guanilo ME, Farina Junior JA, Rossi LA (2010). Burns in the domestic environment: characteristics and circumstances of accidents. Rev Lat Am Enfermagem.

[CR38] Vilasco B, Bondurand A (1995). Burns in Abidjan, Cote d'Ivoire. Burns.

[CR39] WHO. Burns. 2018. Geneva, Switzerland. https://www.who.int/news-room/fact-sheets/detail/burns [accessed 25 February 2022]

[CR40] Wong JM, Nyachieo DO, Benzekri NA (2014). Sustained high incidence of injuries from burns in a densely populated urban slum in Kenya: an emerging public health priority. Burns.

